# Failure to obtain admission sputum culture is associated with higher mortality and fewer ventilator-free days for intubated pneumonia patients: a quality improvement project

**DOI:** 10.1186/cc13451

**Published:** 2014-03-17

**Authors:** Y Mahal, K Smith, R Riker

**Affiliations:** 1 Maine Medical Center, Portland, ME, USA

## Introduction

The primary objective was to assess the impact of failure to obtain sputum culture (SC) among patients requiring intubation for pneumonia. For patients admitted to an ICU with severe pneumonia, guidelines recommend obtaining a lower respiratory tract sample for culture. Our experience suggested this is rarely ordered from the emergency department (ED).

## Methods

We retrospectively reviewed charts of all patients admitted through the ED with a diagnosis of pneumonia requiring intubation in the first 24 hours between January 2011 and November 2012. Patients were classified as SC collected or not collected. We recorded demographic data, SC results, antibiotic choice and de-escalation, ventilator-free days (up to day 14), and mortality in ICU and hospital. Inferential statistics were performed using SPSS version 20.0, with *P *< 0.05 considered significant.

## Results

Of 50 patients we reviewed, 43 (86%) were intubated in the ED, 45 had SC ordered (only eight (18%) by ED physicians), and 37 (74%) had SC collected. There was no difference in age, gender or severity of illness as measured by APACHE score between the two groups. ICU mortality was lower in the SC collected group (24% vs. 69%, *P *= 0.007), as was hospital mortality (30% vs. 77%, *P *= 0.007) and antibiotics were de-escalated more often (89% vs. 8%, *P *< 0.001). Patient with SC collected showed a trend toward significantly more ventilator-free days (6.5 vs. 0, *P *= 0.053).

## Conclusion

Sputum cultures were rarely ordered by ED physicians, and when not obtained in intubated patients with pneumonia, ICU and hospital mortality was higher, there was less antibiotic de-escalation, and a trend toward fewer ventilator-free days. Efforts to improve collection of sputum cultures in these patients are warranted.

